# Feature Selection in High Dimensional Biomedical Data Based on BF-SFLA

**DOI:** 10.3389/fnins.2022.854685

**Published:** 2022-04-18

**Authors:** Yongqiang Dai, Lili Niu, Linjing Wei, Jie Tang

**Affiliations:** ^1^School of Information Science and Technology, Gansu Agricultural University, Lanzhou, China; ^2^School of Food Science and Engineering, Gansu Agricultural University, Lanzhou, China

**Keywords:** feature selection, shuffled frog leaping algorithm, classification accuracy, bacterial foraging algorithm, biomedical data

## Abstract

High-dimensional biomedical data contained many irrelevant or weakly correlated features, which affected the efficiency of disease diagnosis. This manuscript presented a feature selection method for high-dimensional biomedical data based on the chemotaxis foraging-shuffled frog leaping algorithm (BF-SFLA). The performance of the BF-SFLA based feature selection method was further improved by introducing chemokine operation and balanced grouping strategies into the shuffled frog leaping algorithm, which maintained the balance between global optimization and local optimization and reduced the possibility of the algorithm falling into local optimization. To evaluate the proposed method’s effectiveness, we employed the K-NN (k-nearest Neighbor) and C4.5 decision tree classification algorithm with a comparative analysis. We compared our proposed approach with improved genetic algorithms, particle swarm optimization, and the basic shuffled frog leaping algorithm. Experimental results showed that the feature selection method based on BF-SFLA obtained a better feature subset, improved classification accuracy, and shortened classification time.

## Introduction

Biomedical datasets provide the basis for medical diagnostics and scientific research, and feature subset selection was an important data mining method in many application areas (Lu and Han, 2003). Such datasets were generally characterized by high-dimensionality, multiple classes, useless data, and a very lot of features, many of which had weak correlation or independence to corresponding diagnostic or research problems (Misra et al., 2002). Moreover, there may be features (in biomedical datasets) that exhibit a weak correlation with specific diagnostic or research problems. The recognition of the optimal feature subsets can eliminate redundant information and reduce the computational cost required for data mining while improving classification accuracy (Vergara and Estévez, 2014). Feature selection can enhance classification accuracy and decrease the computational complexity in classification. The feature subset should be indispensable and sufficient to describe the target concept while maintaining suitably high precision in the representing the original features.

Effective identification and selection of candidate subsets require an effective and efficient search method and learning algorithm. However, developing such approaches and learning algorithms to identify optimal subsets remains an open research issue. This manuscript proposed a method for enabling feature selection from high-dimensional biomedical data based on the Bacterial Foraging–Shuffled Frog Leaping Algorithm (BF-SFLA).

The BF-SFLA was developed by introducing the convergence factor of the Bacterial Foraging Algorithm (BFA) into the Shuffled Frog Algorithm (SLFA), which was discussed in detail in later sections of this manuscript.

We have used *K*-*NN* and *C*4.5 Decision Tree Classification Method combined with high-dimensional biomedical data to evaluate the BF-SFLA, including performing a comparative analysis of improvement Genetic Algorithm (IGA), improvement Particle Swarm Optimization (IPSO), and the SFLA. The experimental results showed that the feature selection based on BF-SFLA demonstrates better performance in identifying relevant subsets with higher classification accuracy than the alternative methods.

The structure of this manuscript was as follows: the related research was considered in Section II. The BF-SFLA was presented in Section III with the analysis of improvement strategy in Section IV. In Section V, we discussed the application of feature selection. This manuscript ended with Section VI, in which we provide concluding comments.

## Related Research

There were many feature selection algorithms documented in the literature (Wang et al., 2007). A memetic feature selection algorithm was proposed in Lee and Kim (2015) for multi-label classification, preventing premature convergence and improving efficiency. The proposed method employs a memetic procedure to refine the feature subsets found obtained by a genetic search, which improves multi-label classification performance. Empirical studies using a variety of tests indicate the proposed method was superior to the conventional multi-label feature selection methods.

A novel algorithm was proposed in Wang et al. (2017) based on information theory called the Semi-supervised Representatives Feature Selection (SRFS) algorithm. The SRFS was independent of any algorithm learning classification. It can quickly and effectively identify and remove unnecessary information with irrelevant and redundant features. More critical, the unlabeled data were used as the labeled data in the Markov blanket through the correlation gain. The results on several benchmark datasets show that SRFS can significantly improve existing supervised and semi-supervised algorithms.

Li et al. (2015) aim to introduce a new method to stable feature selection algorithms. The experiments used open source “actual microarray data,” challenging for high-dimensional minor sample problems. The reported results indicate that the proposed integrated FREE was stable and has better (or at least comparable) accuracy than was the case for some other commonly stable feature weighting methods.

Tabakhi et al. (2014) proposed an unsupervised feature selection method based on ant colony optimization, which was called UFSACO. In this method, the optimal feature subset was found through multiple iterations without using any learning algorithm(s). UFSAC can be classified as a filter-based multivariate approach. The proposed method has low computational complexity. Therefore, it can be applied to high-dimensional data sets. By comparing the performance of UFSACO with 11 famous univariate and multivariate feature selection methods using different classifiers (support vector machine, decision tree, and Bayes), the experimental results of several commonly used data sets show the efficiency and effectiveness of the UFSACO method and the relevant improvements in the past.

AbdEl-Fattah Sayed et al. (2016) proposed a new hybrid algorithm, which combines the Clonal Selection Algorithm (CSA) with the Flower Pollination Algorithm (FPA) to form Binary Clonal Flower Pollination Algorithm (BCFA), aiming at solving the problem of feature selection. The Optimum-Path Forest (OPF) classification accuracy was taken as the objective function. Experimental testing has been carried out on three public datasets. The reported results demonstrate that the proposed hybrid algorithm achieved striking results compared with other famous algorithms, such as the Binary Cuckoo Search Algorithm (BCSA), the Binary Bat Algorithm (BBA), the Binary Differential Evolution Algorithm (BDEA), and the Binary Flower Pollination Algorithm (BFPA).

Shrivastava et al. (2017) compared and analyzed various nature-inspired algorithms to select the optimal features required to help in the classification of affected patients from the population. The reported experimental results show that the BBA outperformed traditional techniques such as Particle Swarm Optimization (PSO), Genetic Algorithms (GA), and the Modified Cuckoo Search Algorithm (MCSA) with a competitive recognition rate for the selected features dataset.

Zhang et al. (2015) suggested a new method using the Bones Particle Swarm Optimization (BPSO) to find the optimal feature subset, which was termed the binary BPSO. In this algorithm, a reinforcement memory strategy was designed to update the local “leaders” of particles to avoid the degradation of excellent genes in particles. A uniform combination was proposed to balance the local exploitation and the global mining of the algorithm. In addition, the 1-nearest neighbor method was used as a classifier to evaluate the classification accuracy of particles. The proposed algorithm was evaluated by several international standard datasets. Experimental testing shows that the proposed algorithm has strong competitiveness in classification accuracy and computational performance.

Based on the concept of decomposition and fusion, a practical feature selection method for large-scale hybrid datasets was proposed by Wang and Liang (2016) to identify an effective feature subset in a short time. By using two common classifiers as evaluation functions, experiments have been performed on 12 UCI data sets. The result of the experiment showed that the proposed method was effective and efficient.

Cai et al. (2020, 2021) aimed to construct a novel multimodal model by fusing different electroencephalogram (EEG) data sources, which were under neutral, negative and positive audio stimulation, to discriminate between depressed patients and normal controls. Then, from the EEG signals of each modality, linear and nonlinear features were extracted and selected to obtain features of each modality.that the fusion modality could achieve higher depression recognition accuracy rate compared with the individual modality schemes. This study may provide an similarity between features, which leads to minimizing the redundancy. As a result, it could be classified as a filter-based multivariate approach. The proposed approach has low computational complexity. Therefore, it was suitable for high-dimensional data sets.

The relevant research shows that nature incentive systems represent a practical basis for feature selection. In this manuscript, we have applied nature-inspired method using our new extended SFLA (the BF-SFLA) for high-dimensional biomedical data feature selection.

## The Proposed Based on the Chemotaxis Foraging-Shuffled Frog Leaping Algorithm

### The Shuffled Frog Leaping Algorithm

**FIGURE 1:**
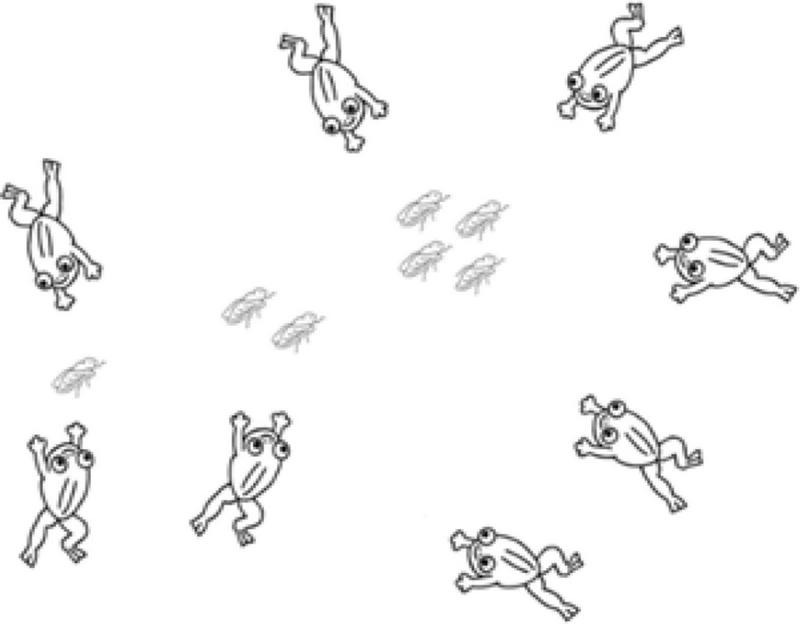
The simulation diagram of biological characteristics of SFLA.


The biological characteristics of the SFLA are shown in Figure 1. It could be seen from the figure that a large number of individual frogs were distributed in the search space, and there were several food-dense areas (extremal points of the function). The individuals were assigned to several groups based on the fitness (from big/small to small/big). The algorithm update strategy is shown in Equations (1) and (2), in which the worst individual (*P*_*w*_) learned from the best individual (*P*_*b*_) of the subgroup. Without progress, (*P*_*w*_) would learn from the global best individual (*P*_*g*_). If there was still no progress, (*P*_*w*_) would be replaced by random individuals. The number of iterations in the algorithm was given by (t). Where: 1) *P*_*w*_(*t*+1) was a new individual generated by the updating strategy, 2) *D*(*t*+1) was the length of each moving step, and 3) *R* was a random number with a change range of [0, 1].

(1) $$D\,\left(t+1\right)=R\times \left(P_{b}-P_{w}\right)$$

(2) $$P_{w}\,\left(t+1\right)=P_{w}\,\left(t\right)+D\,\left(t+1\right)$$

Following updating, if the newly generated *P*_*w*_(*t*+1) was better than the old *P*_*w*_(*t*), *P*_*w*_(*t*) would be replaced by *P*_*w*_(*t*+1). Otherwise, (*P*_*b*_) would be replaced by (*P*_*g*_). If (*P*_*w*_) was still not improving, it would be randomly replaced by a new individual. This iterative process with the number of iterations was equal to the number of subgroup individuals. When the subgroup processing was completed, all subgroups would be randomly sorted and reclassified into new subgroups. The process was repeated until the pre-determined termination conditions were satisfied.

The SFLA was one of many nature-inspired algorithms based on swarm intelligence (Eusuff and Lansey, 2003). It has the following characteristics: (1) a simple concept, (2) reduced parameters, (3) strong performance optimization, (4) fast calculation speed, and (5) easy implementation. It has been widely used in many fields such as model recognition problems (Shahriari-kahkeshi and Askari, 2011; Hasanien, 2015), scheduling problems (Pan et al., 2011; Alghazi et al., 2012), parameter optimization problems (Perez et al., 2013), traveling salesman problem (Shrivastava et al., 2017), unit commitment problem (Ebrahimi et al., 2012), distribution problem (Gomez Gonzalez et al., 2013), and the controller problem (Huynh and Nguyen, 2009).

### The Bacterial Foraging Algorithm

Through simulation, *E. coli* ate food in the human intestinal tract. The Bacterial Foraging Algorithm (referred to as BFA) (Passino, 2002) was proposed in 2002 by Passino et al., and because the BFA has shown improved optimization performance, it has attracted significant research by scholars in the field. The BFA included three steps, Chemokines Operation (referred to as CO), Propagation Operation (referred to as PO), and Dissipation Operation (Referred to as DO), and the (CO) was the core step.

The (CO) corresponds to the direction selection strategy adopted by bacteria in searching for food, which played a significant role in the algorithm’s convergence. In the process of (CO), the motion mode of bacteria could be divided into two states: Rotation and Forward. The Rotating motion mode refers to the operation of the moving unit step after the bacteria changes the direction. In contrast, the Forward motion mode refers to that after the bacteria complete the rotating motion; if the quality of the solution was improved, the bacteria would continue to move several steps in the same direction until the adaptive value of the function did not change, or the predetermined number of moving steps was reached.

### The Shuffled Frog Leaping Algorithm Based on Chemotactic Operation

#### Proposed Improvements

In the SFLA, the worst individual (*P*_*w*_) from a subgroup learned to form the optimal individual (*P*_*b*_) in the same subgroup or the optimal global individual (*P*_*g*_) iteratively. IF the fitness was not improved in this process, a randomly generated new individual replaced the existing (*P*_*w*_), while maintaining population diversity may result in the failure to identify potentially more optimal solutions. This result was because following the (*P*_*w*_) learned from (*P*_*b*_) or (*P*_*g*_), while partial improvement (in the fitness) may have been achieved, there may be better solutions in the neighborhood if the new randomly generated individual was used in place of the existing (*P*_*w*_). The possibility of finding a better solution was lost by the SFLA. Inspired by the (CO) of the BFA, this manuscript introduced the (CO) into SFLA and guided (*P*_*w*_) to refine the search in the neighborhood and find better solutions.

#### Proposed Updating Strategy

Section (III B), considered Rotation and Progression. Our updating strategy proposed that (*P*_*w*_) moved stepwise in random directions (in the solution space) and completed the rotation operation. IF the fitness was improved, (*P*_*w*_) would move forward in the same direction repeatedly until the fitness no longer was improved, at which point (*P*_*w*_) would be replaced by a random individual in the solution space. The chemotaxis operation strategy was used in a secondary process to increase the granularity of the solution space exploration. This processed secondary aims to search for the potential optimal solution(s) in the (*P*_*w*_) neighborhood, expand the individual search level, improve the local search ability, and improve the search accuracy of the algorithm while maintaining the population diversity.

Of course, when (*P*_*w*_) learned from (*P*_*b*_) and (*P*_*g*_) without progress, the (CO) was not always performed on every iteration. To strengthen space exploration ability at the early stage of the iteration, the algorithm must keep specific diversity, so the (CO) was used with less probability, and in the middle and later stages of the iteration, to strengthen the optimal neighborhood mining density, the algorithm must improve the local searchability. To balance the relationship between algorithm exploration and mining, the curve change formula was introduced to calculate the (CO) perform probability.


(3) $$a=\text{exp}\,\left(-30\times {\left(\frac{g}{G}\right)}^{s}\right)$$

(4) $$C=\left\{ \begin{array}{c} 1if\left(a<R\right)\\ 0if\left(a\geq R\right) \end{array}\right.$$

**FIGURE 2:**
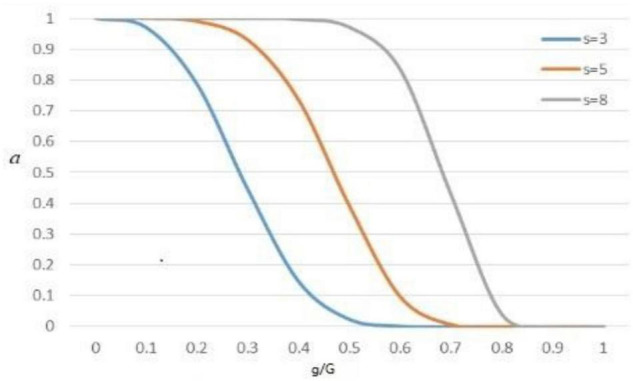
The curve of function *a.*

The function (a) was calculated by Equation (3), where (g) was current iteration number and (G) was total iteration number. Figure 2 was the graph of the value of function a when (s) was equal to 3, 5, and 8, respectively. To balance the relationship between the algorithm exploration and mining, (s) was set as 5 in subsequent experiments. (R) was the random number between [0, 1]. C was the decision factor in Equation (4), if C was 1 perform the (CO), and if *C* was 0 do not perform the (CO).

### The Improvement of Grouping Strategy

The grouping strategy of the SFLA was as follows: suppose that *P* individuals were sorted into *m* groups according to the quality of the solution (function evaluation value), and *n* groups were divided into each group, where *P* = m*n. Then the *first* individual, the *m*+1 individual, …, the (*n*–1)*m+1 individual, was assigned to the 1st group. The *second* individual, the *m*+2 individuals…, the (*n*–1)*m+2 individuals were assigned to the second group, and so on, the *mth* individual, the 2*m* individual…, the nth*m individuals were assigned to the group. Until all the individuals were grouped, this grouping strategy was called Classic Grouping Strategy (CGS).

To verify the contribution of CGS to the global optimal solution *P*_*g*_, 15 standard test functions were used for the simulation experiment. The parameters of the test function were shown in Table 1. Test function parameters and target accuracy information were shown in Table 1. The average value of the algorithm ran independently 30 times was used for the experimental data. Algorithm parameters were set as follows: total population, 200; number of groups, 10; individual in a subgroup, 20; number of updates and evolution within subgroup, 20; number of iterations of the algorithm, 500. The operating environment of the algorithm was Windows 10 operating system, 8-core 64-bit processor and 8G memory, and the running software was MATLAB2012 a.

**TABLE 1:** Parameters of the benchmark function.

Function	Dimensions(n)	Scope	Optimal value	Accuracy
${\text{f}}_{1}\,\left(\text{x}\right)=\sum_{\text{i}=1}^{\text{n}}{\text{x}}_{\text{i}}^{2}$	30/60/90	[–5.12,5.12]	0	| Actual Value –0| < 1 × 10^–16^
${\text{f}}_{2}\,\left(\text{x}\right)=\sum_{\text{i}=1}^{n-1}\left(100\,{\left({\text{x}}_{\text{i}+1}^{2}-{\text{x}}_{\text{i}}\right)}^{2}+{\left(1-{\text{x}}_{\text{i}}\right)}^{2}\right)$	30/60/90	[–30,30]	0	| Actual Value –0| < 1 × 10^1^
${\text{f}}_{3}\,\left(\text{x}\right)=\sum_{\text{i}=1}^{\text{n}}\left({\text{x}}_{\text{i}}^{2}-10\,c\,o\,s\,\left(2\,{\text{π}\text{ x}}_{\text{i}}\right)+10\right)$	30/60/90	[–5.12,5.12]	0	| Actual Value –0| < 1 × 10^1^
${\text{f}}_{4}\,\left(\text{x}\right)=\frac{1}{4000}\,\sum_{\text{i}=1}^{\text{n}}{\text{x}}_{\text{i}}^{2}-\prod_{\text{i}=1}^{\text{n}}\cos\left(\frac{{\text{x}}_{\text{i}}}{\sqrt{\text{i}}}\right)+1$	30/60/90	[–600,600]	0	| Actual Value –0| < 1 × 10^–2^
${\text{f}}_{5}\,\left(\text{x}\right)=-20\,\exp\left(-0.2\,\sqrt{\frac{1}{\text{n}}\,\sum_{\text{i}=1}^{\text{n}}{\text{x}}_{\text{i}}^{2}}\right)-\exp\left(\frac{1}{\text{n}}\,\sum_{\text{i}=1}^{\text{n}}\mathrm{cos2}\,\pi \,x_{\text{i}}\right)+20+\text{e}$	30/60/90	[–32,32]	0	| Actual Value –0| < 1 × 10^–7^
${\text{f}}_{6}\,\left(\text{x}\right)=\sum_{\text{i}=1}^{n-1}{\left({\text{x}}_{\text{i}}^{2}+{\text{x}}_{\text{i}+1}^{2}\right)}^{0.25}\,\left[\sin^{2}\left(50\,{\left({\text{x}}_{\text{i}}^{2}+{\text{x}}_{\text{i}+1}^{2}\right)}^{0.1}\right)+1\right]$	30/60/90	[–100,100]	0	| Actual Value –0| < 1 × 10^0^
${\text{f}}_{7}\,\left(\text{x}\right)=\sum_{\text{i}=1}^{\text{n}}\left|{\text{x}}_{\text{i}}\right|+\prod_{\text{i}=1}^{\text{n}}\left|{\text{x}}_{\text{i}}\right|$	30/60/90	[–10,10]	0	| Actual Value –0| < 1 × 10^–16^
f_8_(x) = Max{|x_i_|}	30/60/90	[–100,100]	0	| Actual Value –0| < 1 × 10^–2^
${\text{f}}_{9}\,\left(\text{x}\right)=\sum_{\text{i}=1}^{\text{n}}\mathrm{int}\,{\left({\text{x}}_{\text{i}}+0.5\right)}^{2}$	30/60/90	[–100,100]	0	| Actual Value –0| < 1 × 10^–16^
${\text{f}}_{10}\,\left(\text{x}\right)=\sum_{\text{i}=1}^{\text{n}}{\text{ix}}_{\text{i}}^{4}+\text{r}$*andom*(0,1]	30/60/90	[–1.28,1.28]	0	| Actual Value –0| < 1 × 10^–3^
${\text{f}}_{11}\,\left(\text{x}\right)=-\sum_{\text{i}=1}^{\text{n}}{\text{x}}_{\text{i}}\,\text{sin}\,\left(\sqrt{\left|{\text{x}}_{\text{i}}\right|}\right)$	30/60/90	[–500,500]	–418.9829*n*	| Actual Value –(–418.9829*n*) | < 1 × 10^2^
${\text{f}}_{12}\,\left(\text{x}\right)=\frac{\Pi }{\text{n}}\,\left\{10\,\sin^{2}\left(\Pi \,y_{\text{i}}\right)+\sum_{\text{i}=1}^{n-1}{\left({\text{y}}_{\text{i}}-1\right)}^{2}\,\left[1+10\,{\text{sin}}^{2}\,\left(\Pi \,y_{\text{i}+1}\right)\right]+{\left({\text{y}}_{\text{n}}-1\right)}^{2}\right\}+\sum_{\text{i}=1}^{\text{n}}\text{u}\,\left({\text{x}}_{\text{i}},10,100,4\right)$				
${\text{y}}_{\text{i}}\text{ = }1+\frac{{\text{x}}_{\text{i}}+1}{4},\text{ u}\left({\text{x}}_{\text{i}},\text{a},\text{k},\text{m}\right)\text{ = }\left\{\begin{array}{c} \text{ k}{\left({\text{x}}_{\text{i}}-\text{a}\right)}^{\text{m}}{\text{ x}}_{\text{i}}\text{ > a}\\ 0\;-\text{a }\leq {\text{x}}_{\text{i}}\;\leq \text{a}\\ \text{k}{\left(-{\text{x}}_{\text{i}}-\text{a}\right)}^{\text{m}}{\text{ x}}_{\text{i}}\text{ < }-\text{a} \end{array}\right\}$	30/60/90	[–50,50]	0	| Actual Value –0| < 1 × 10^–15^
${\text{f}}_{13}\,\left(\text{x}\right)=4\,x_{1}^{2}-2.1\,{\text{x}}_{1}^{4}+\frac{{\text{x}}_{1}^{6}}{3}+{\text{x}}_{1}\,{\text{x}}_{2}-4\,x_{2}^{2}+4\,x_{2}^{4}$	2	[–5,5]	–1.0316285	| Actual Value –1.0316285)| < 1 × 10^–3^
${\text{f}}_{14}\,\left(\text{x}\right)={\left({\text{x}}_{2}-\frac{5.1}{4\,\pi^{2}}\,{\text{x}}_{1}^{2}+\frac{5}{\pi }\,{\text{x}}_{1}-6\right)}^{2}+10\,\left(1-\frac{1}{8}\right)\,{\text{cosx}}_{1}+10$	2	[–15,15]	0.398	| Actual Value –(0.398)| < 1 × 10^–2^
${\text{f}}_{15}\,\left(\text{x}\right)=\frac{{\text{sin}}^{2}\,\sqrt{{\text{x}}_{1}^{2}+{\text{x}}_{2}^{2}}-0.5}{{\left[1+0.001\,\left({\text{x}}_{1}^{2}+{\text{x}}_{2}^{2}\right)\right]}^{2}}-0.5$	2	[–100,100]	–1	| Actual Value –(–1)| < 1 × 10^–4^

**FIGURE 3:**
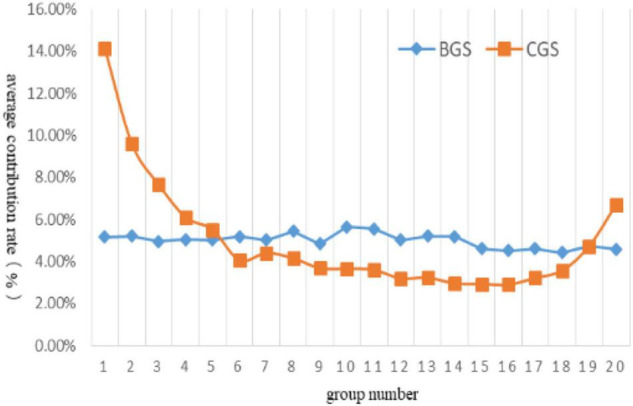
The average contribution rate of each group updating *P*_*g*_.

The experimental results were shown in Figure 3. In the figure, the abscissa represented the group number, and the ordinate represented the average contribution rate of each group updating *P*_*g*_. It could be seen from the figure that, compared with other groups, group 1 to group 5 obtained a higher average update contribution rate to *P*_*g*_, among which group 1 obtained the highest contribution rate (14.11%), and the total contribution rate of the five groups was 43.00%.

According to the CGS grouping strategy, the individuals with a higher quality of each equilateral solution were first assigned to the groups with smaller numbers. The smaller the group number, the higher the quality of the assigned solution would be. The individual quality of groups with smaller group numbers was better than groups with more significant group numbers. In the process of algorithm operation, if these grouping individuals once fell into the local optimal, because the update of *P*_*g*_ was highly dependent on these groups, it would be difficult to rely on other groups with low contribution rate to *P*_*g*_ to guide the algorithm to jump out of the local optimal, thus increasing the probability of the algorithm falling into the local optimal overall. To avoid this situation, it was necessary to balance the contribution proportion of each group to *P*_*g*_, reduce the dependence of *P*_*g*_ update on specific groups, and improve the ability to jump out after the algorithm fell into the local optimal.

#### Improved Grouping Strategy

1 to *m* individuals were assigned to each group in sequence (1) in each group, the *m*+1 to 2*m individuals according to the reverse was assigned to each group (1) in each group, then the 2*m+1 to 3*m individuals were assigned to each group by the order again (1) in each group, the 3*m+1 to 4*m individuals according to the reverse was assigned to each group (1) in each group, and so on, until all the individual were grouped.

The improved grouping strategy could effectively avoid the individuals with better quality of solutions into the same group and guarantee the average solution quality of individuals in each group. In this way, the proportion of each group’s contribution to the optimal global solution could be effectively balanced, thus reducing the possibility of the algorithm falling into the local optimal. This grouping strategy was called Balance Grouping Strategy (BGS).

## The Analysis of Improvement Strategy

After (CO) was introduced into the SFLA, the balance between Exploratory Search in the early stage and Refined search in the later stage of the algorithm iteration were well handled, the SFLA with a single introduction of (CO) was named as SFLA1. The contribution of (BGS) was to balance the update contribution rate of groups for the global best individual (*P*_*g*_) and avoid the SFLA falling into the local optimization. The SFLA with a single (BGS) was named SFLA2.

(CO) and (BGS) were two improved strategies of SFLA. Among them, the former was the improvement of the updating method for the worst individuals, and the latter was the optimization of the algorithm grouping method. Although one kind of single improvement strategy could improve the optimization performance of the algorithm to a certain extent, the improvement effect was limited. However, the performance improvement of the algorithm would be more evident if the two improvement strategies were combined. (CO) and (BGS) were all introduced into the SFLA simultaneously. The improved algorithm was named Bacterial Foraging-Shuffled Frog Leaping Algorithm, referred to as BF-SFLA.

To verify the actual optimization performance of SFLA1, SFLA2, and BF-SFLA, 15 standard test functions were selected for verification experiments. The Parameter Settings of test functions were shown in Table 1. The algorithms parameters were set as follows: the total population was 400. The subgroups number was 40. The number of individuals in each subgroup was 10. The number of updating evolution within every subgroup was 10. The number of algorithm evolution was 500. The experimental results were shown in Table 2. The operating environment was Windows 10, 8-core 64-bit operating system with 8G of memory, and the running software was MATLAB 2012a.

**TABLE 2:** The experimental results under fixed iteration number.

*Function*	SFLA		SFLA1		SFLA2		SFLA^[25]^		SFLA^[26]^		BF-SFLA	
	Ave	Std	Ave	Std	Ave	Std	Ave	Std	Ave	Std	Ave	Std
f_1_	9.36E–01	8.66E–02	1.47E–33	4.92E–20	9.05E–01	6.68E–02	6.45E–03	3.12E–03	5.22E–03	7.32E–33	3.21E–18	5.02E–33
f_2_	1.46E+02	6.59E+01	2.54E+01	1.71E+01	1.01E+02	6.08E+01	2.67E+02	5.28E+01	1.29E+02	3.05E–01	2.57E+01	4.63E–01
f_3_	1.59E+01	4.39E+00	1.03E+00	3.19E+00	1.30E+01	4.11E+00	1.95E+01	7.07E+00	1.16E+01	1.56E+00	8.73E+00	2.03E+00
f_4_	1.09E+00	4.57E–02	1.00E+00	1.60E–16	1.04E+00	3.11E–02	1.00E+00	2.14E–04	1.00E+00	1.93E–16	1.00E+00	2.14E–16
f_5_	1.41E+00	5.68E–01	1.06E–14	2.62E–12	1.07E+00	5.26E–01	1.09E+00	6.62E–01	7.50E–01	3.18E–15	1.12E–12	7.68E–15
f_6_	2.44E+01	7.33E+00	1.91E–01	3.37E+00	2.27E+01	8.54E+00	1.91E+01	4.46E+00	1.69E+01	2.88E–01	6.05E+00	3.88E–01
f_7_	1.01E+00	1.08E–01	1.14E–17	6.22E–35	9.68E–01	3.66E–02	5.99E–01	1.50E–01	3.11E–01	2.66E–17	1.14E–35	2.77E–18
f_8_	6.62E+00	9.98E–01	3.32E–04	3.53E–01	4.06E+00	9.44E–01	5.01E+00	7.40E–01	4.32E+00	2.54E–04	1.08E+00	4.47E–04
f_9_	0.00E+00	0.00E+00	0.00E+00	0.00E+00	0.00E+00	0.00E+00	0.00E+00	0.00E+00	0.00E+00	0.00E+00	0.00E+00	0.00E+00
f_10_	5.18E–01	1.57E–01	1.02E–03	8.29E–04	5.02E–01	9.63E–02	2.16E–03	8.03E–04	2.90E–03	3.30E–04	2.41E–03	3.99E–04
f_11_	–3.05E+03	4.01E+02	–4.61E+03	6.48E+02	–3.01E+03	4.20E+02	–5.09E+03	5.67E+02	–4.77E+03	3.55E+02	–4.94E+03	2.48E+02
f_12_	9.30E–01	6.60E–02	1.92E–32	7.25E–15	8.09E–01	8.40E–02	4.90E–02	7.51E–02	5.74E–02	2.06E–33	1.11E–17	1.40E–32
f_13_	–7.68E–01	2.01E–01	–1.03E+00	2.51E–04	–7.87E–01	2.53E–01	–1.03E+00	0.00E+00	–1.03E+00	1.05E–03	–1.03E+00	9.72E–04
f_14_	3.98E–01	1.70E–01	3.98E–01	0.00E+00	3.98E–01	1.78E–01	3.98E–01	3.98E–01	3.98E–01	0.00E+00	3.98E–01	0.00E+00
f_15_	–8.77E–01	6.40E–02	–1.00E+00	3.36E–03	–8.63E–01	7.23E–02	–1.00E+00	0.00E+00	–9.98E–01	2.69E–04	–1.00E+00	7.35E–04

Two modes, (1) the algorithm optimization accuracy analysis under fixed iterations number and (2) the algorithm iterations number analysis under the fixed optimization accuracy, were used to evaluate the optimization performance of the algorithm.

(1) The algorithm optimization accuracy analysis under fixed iterations number

The experimental results were analyzed with the algorithm optimization accuracy under fixed iterations number, as shown in Table 2. Where (Ave) represented the average optimal value of the algorithm running 30 times, (Std) represented the standard deviation, and (AvgT(s)) represented the average running time each time, in seconds (s). The following results could be obtained from Table 2:

(1) For all test functions (F1 to F15), SFLA1 and SFLA2 obtained better (Ave) and (Std) than SFLA to varying degrees, indicating that the two improvement strategies all played a specific role in improving the performance of the algorithm. Compared with the SFLA, the (Ave) of SFLA1 and SFLA2 had been improved by E^0^ to E^10^, and the (Std) had been reduced by E^0^ to E^20^, indicating that the improved strategies of SFLA1 and SFLA2 played more pronounced effects on improving the optimization accuracy and stability of the algorithm.

(2) For all test functions, BF-SFLA obtained more minor (Ave) and (Std) compared with SFLA1 and SFLA2 to varying degrees, indicating that the optimization accuracy and stability of the algorithm after the introduction of the combined improvement strategies were better than single improvement strategy. SFLA1 and SFLA2 were two algorithms obtained by SFLA after introducing (CO) and (BGS), respectively. (CO) was the improvement of updating method for (*P*_*w*_), while (BGS) was the optimization for algorithm grouping method. Although a single improvement strategy could improve the optimization performance of the algorithm to a certain extent, the room for improvement was limited. However, by combining multiple improvement strategies and improving the algorithm from different perspectives, the performance improvement of the algorithm would be more obvious. Compared with the improved algorithms in literature (Sun et al., 2008) and (Dai and Wang, 2012), BF-SFLA had obtained better (Ave) for almost all test functions (except f_10_). On the whole, it showed that BF-SFLA had better optimization accuracy and performance.

(2) The algorithm iterations number analysis under the fixed optimization accuracy

The SFLA, SFLA1, SFLA2, Improved SFLA in literature (Sun et al., 2008; Dai and Wang, 2012), and BF-SFLA were used to optimize and verify the test function, verify the iteration conditions of six algorithms independently executing 30 times (the maximum number of iterations being 500) to meet the accuracy requirements in Table 1. The relevant information was shown in Table 3. In the table, (Avg(%)) represented the success rate (the percentage of the number of experiments where the algorithm achieved the required accuracy in the total number of experiments). (AveN) represented the average number of iterations with the required accuracy. The following results can could be obtained from Table 3.

**TABLE 3:** The experimental results under fixed optimization accuracy.

*Function*	SFLA		SFLA1		SFLA2		SFLA^[25]^		SFLA^[26]^		BF-SFLA	
	Ave(%)	AveN	Ave(%)	AveN	Ave(%)	AveN	Ave(%)	AveN	Ave(%)	AveN	Ave(%)	AveN
f_1_	0%	–	23%	407	0%	–	100%	261	100%	283	100%	248
f_2_	0%	–	93%	298	0%	–	100%	121	97%	260	100%	94
f_3_	23%	385	100%	145	43%	306	100%	140	47%	295	100%	126
f_4_	0%	–	90%	201	0%	–	97%	149	80%	339	93%	138
f_5_	0%	–	100%	267	0%	–	100%	266	0%	–	100%	249
f_6_	3%	482	100%	208	7%	437	100%	192	7%	424	100%	182
f_7_	0%	–	100%	344	0%	–	0%	–	0%	–	100%	342
f_8_	0%	–	100%	120	0%	–	0%	–	0%	–	100%	120
f_9_	100%	128	100%	23	100%	127	100%	65	100%	76	100%	20
f_10_	100%	93	100%	32	100%	72	100%	23	100%	119	100%	26
f_11_	63%	231	93%	66	73%	220	63%	220	70%	231	100%	170
f_12_	0%	–	93%	232	0%	–	0%	–	0%	–	97%	192
f_13_	70%	148	100%	31	40%	144	100%	72	100%	59	100%	77
f_14_	100%	20	100%	16	100%	20	100%	19	100%	16	100%	15
f_15_	77%	220	80%	133	87%	217	87%	182	100%v	117	100%	74

*The symbol “–” indicates that the fixed optimization accuracy cannot be achieved within the 500 times.*

(1) SFLA had a success rate of 0% for test functions f_1_, f_2_, f_4_, f_5_, f_7_, f_8_, and f_12_, and could not achieve the required optimization accuracy within a fixed number of iterations (500), indicating that SFLA had a slow convergence speed and low convergence accuracy. Compared with SFLA, SFLA1 and SFLA2 achieved a specific success rate for all test functions, indicating that the algorithm improved by introducing a single strategy improved the convergence accuracy of the algorithm to a certain extent.

(2) The BF-SFLA achieved a success rate of 93–100% for all test functions. The result was significantly higher than the other five algorithms. It showed that BF-SFLA had better-searching precision and stability. From the AveN indexes with fixed optimization accuracy, BF-SFLA was smaller than the other five algorithms on the whole. The results showed that BF-SFLA converges faster and obtains the same optimization precision with fewer iteration times.

Table 4 was the index mean information table under fixed iteration times. Where AVE(Avg) and AVE(Std) were, respectively the means of (Ave) and (Std) for all test functions in Table 2. Compared with SFLA, SFLA1, SFLA2, and literature (Sun et al., 2008; Dai and Wang, 2012), the smaller AVE(Ave) and AVE(Std) were achieved by BF-SFLA, so the better optimization performance was achieved by BF-SFLA. Table 5 was the index mean value under fixed optimization accuracy. Where AVE(ave%) and AVE(AveN) were, respectively the means of (Ave(%)) and (AveN) for all test functions in Table 3. Compared with SFLA, SFLA1, SFLA2, and literature (Sun et al., 2008; Dai and Wang, 2012), the smaller AVE(Ave(%)) and AVE(AveN) were achieved by BF-SFLA, so the better optimization performance was also achieved by BF-SFLA.

**TABLE 4:** The index mean of fixed iteration times.

Attribute	SFLA	SFLA1	SFLA2	SFLA^[25]^	SFLA^[26]^	BF-SFLA
AVE(Ave)	6.48E+02	5.32E+02	6.47E+02	5.20E+02	5.31E+02	**5.11E+02**
AVE(Std)	3.21E+01	4.48E+01	3.30E+01	4.22E+01	2.38E+01	**1.67E+01**

*The best value is in bold.*

**TABLE 5:** The index mean value under fixed optimization accuracy.

Attribute	SFLA	SFLA1	SFLA2	SFLA^[25]^	SFLA^[26]^	BF-SFLA
AVE(Ave(%))	35.73%	91.47%	36.67%	76.47%	57.21%	**99.33%**
AVE(AveN)	323.62	139.85	311.00	217.54	282.77	**133.15**

*The best value is in bold.*

## The Application of Feature Selection Based on BF-SLFA Algorithm

### Discretization of the Shuffled Frog Leaping Algorithm

To represent the feature subset, SFLA should be converted to binary SFLA. Assuming that one solution of the algorithm was (0, 1, 0, 1, 0, 0, 1, 0, 0, 1), then the dimension of the solution was 10, and the matching feature subset was one feature subset composed of four in all ten features (the 2nd, 4th, 7th, and 10th). The transformation formula discussed in Hu and Dai (2018) was shown in formula (3, 4), and new *P*_*w*_ was converted into a vector of binary range [0, 1] by Equation (5, 6):


(5) $$s\,i\,g\,\left(D\right)=\frac{1}{1+e^{-A\times D}}$$

$$A=\frac{g}{G}\,\left(F_{1}-F_{2}\right)+F_{2}$$

(6) $$P_{i}=\left\{ \begin{array}{c} 1if(sig\left(D\right)>R\\ 0if(sig\left(D\right)\leq R \end{array}\right.$$

(*P*_*i*_) was the value of the *i*-dimension after the individual was discrete, (*D*) was the step size of the individual, (*R*) was the random number between [0, 1], and *A* was the adjustment coefficient, reflecting the degree of certainty that the individual linear solution was converted to the discrete solution. The value of (*A*) changed from large to small, the determinacy of the individual linear solution to discrete solution changed from strong to weak, and the diversity of individuals changed from weak to strong. Meanwhile, the global exploration ability of individuals changed from strong to weak, and the local mining ability changed from weak to strong. So the value of *A* was neither bigger nor smaller. The value of *A* was determined by four parameters, namely (*g*) (current iteration number), (*G*) (total iteration number), (*F*_1_) (start control parameter), and (*F*_2_) (end control parameter). It was expected that at the beginning of the iteration, (*A*) should be a large value to enhance the exploration ability of the algorithm to traverse the solution space globally in the early stage of the iteration. In contrast, at the later iteration stage, (*A*) should be a small value to enhance the algorithm’s local refinement searchability. Therefore, the value range of (*F*_1_) was set as [0.90, 0.95], and the value range of (*F*_2_) was set as [1.05, 1.1].

The addition and subtraction operation of the discrete binary solution was basically the same as the binary addition and subtraction operation method. The difference was that the highest bit could be borrowed or carried without recording to ensure that the number of elements of the solution vector was consistent with the original number of features. The specific operation was shown in Table 6.

**TABLE 6:** Addition and subtraction of discrete binary solutions.

X^1^	X^2^	X^1^-X^2^	X^1^+X^2^
(1, 0, 1, 0)	(0, 1, 0, 0)	(0, 1, 1, 0)	(1, 1, 1, 0)

### Algorithm Flow

The algorithm flow of the feature selection application based on BF-SFLA was as follows.

Step 1: Set the relevant parameters: (i) randomly generate (*L*) frogs within the scope of the domain, (ii) the number of subgroups was (*A*), (iii) the number for each subgroup frog was (*B*), (iv) the number of global information exchange was *C1*, and (v) the number of local searches was *C2.*

Step 2: Calculate the fitness [value] for each frog. Rank and group all frogs according to the target function value.

Step 3: *IF* (*P*_*w*_) had not been improved after learning from (*P*_*b*_) or (*P*_*g*_), the (CO) would be implemented. *IF* there was no improvement, (*P*_*w*_) was replaced in the solution space by randomly generated individuals.

Step 4: Reorder each subgroup and update (*P*_*w*_), (*P*_*b*_), and (*P*_*g*_) in each subgroup.

Step 5: Determine *IF* the number of local search iterations reaches C2, *IF* not, return to step 3 and continue to execute.

Step 6: Determine *IF* global information exchange iterations reach *C1* or (*P*_*g*_) and *IF* the requirements of convergence precision were achieved. *IF NOT*, return to step 2 to continue. *IF* the termination of the algorithm was reached, output (*P*_*g*_).

**FIGURE 4:**
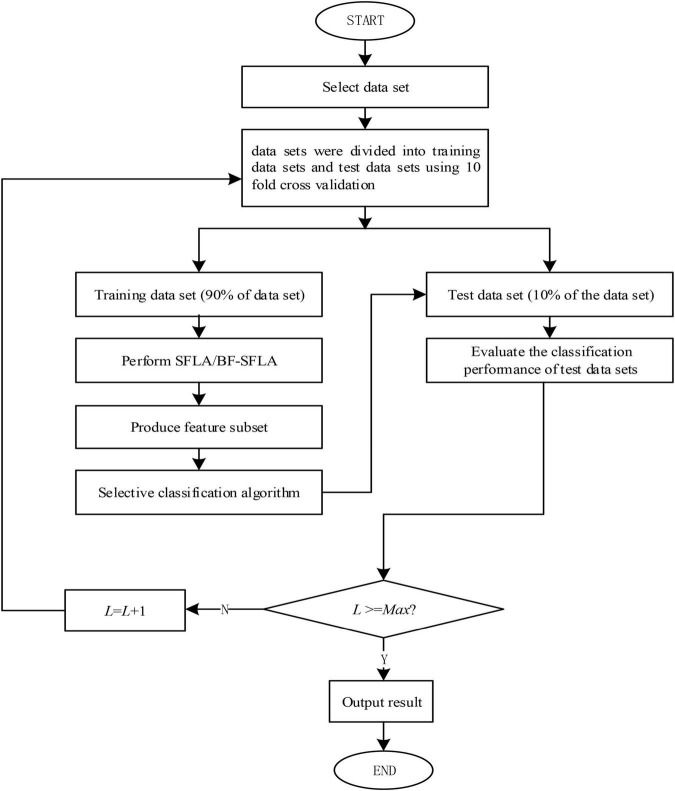
The feature selection flow chart.

The details of the process used for enabling Feature Selection with BF-SFLA were shown in Figure 4; (*L*) was the number of times the algorithm was executed in each experiment, (*D*_*max*_) was the upper limit of feature subsets number, and (*L*_*max*_) was the experiment number.

The classification accuracy and the number of feature subsets were two critical indexes for designing the evaluation function. The classification accuracy was usually obtained by the classification algorithm. *K*-*NN* (k-nearest Neighbor) and *C*4.5 decision tree classification algorithms were used to classify and evaluate the feature subsets without loss of generality.

K-nearest neighbor method was a non-parametric classification technique based on analogy learning. It was very effective in pattern recognition based on statistics, and could achieve high classification accuracy for unknown and non-normal distribution. It had the advantages of robustness and clear concept. The main idea of the *K*-*NN* classification algorithm was as follows: first calculate the distance or similarity between the sample to be classified and the training sample of the known category (usually used Euclidean distance to determine the similarity of the sample), and find the nearest (*K*) neighbors of the distance or similarity with the sample to be classified. Then the category of the sample data to be classified was judged according to the category of the neighbors. If the (*K*) neighbors of the sample data to be classified all belonged to the same category, then the sample to be classified also belonged to the same category. Otherwise, each candidate category was graded to determine the sample data category to be classified according to some rule (Cai et al., 2020).

*C*4.5 decision tree classification algorithm was a greedy algorithm, which adopted a top-down divide and conquer construction. It deduced the classification rules in the form of decision tree representation from a group of unordered and irregular cases, and it was an inductive learning method based on examples. The decision tree classification algorithm was one of the widely used classification algorithms. The advantages of this method were simple description, fast classification speed, and easy-to-understand classification rules.

In our proposed method, the classification accuracy and the number of selected features were the two indicators used to design the evaluation function as defined in Chuang et al. (2008):


(7) $$\text{fitness}={\text{W}}_{1}\times {\text{accW}}_{2}*\left(1-\frac{\text{n}}{\text{N}}\right)$$

The fitness function defined by equation (7) had two predefined weights: (*W*_1_) (the classification accuracy) and (*W*_2_) (the selected feature). If accuracy was the most critical factor, the accuracy [of the weight] could be adjusted to a high value. In this manuscript, the values for (*W*_1_) and (*W*_2_) were (Lu and Han, 2003) and [0.1], respectively. Assuming that an individual with a high fitness [value] had a high probability of including the positions of other individuals in the next iteration, the weights (*W*_1_) and (*W*_2_) must be adequately defined; (*acc*) was the classification accuracy, where (*n*) was the number of unique features and (*N*) was the total number of features.

The fitness definition (*acc*) represented the percentage of correctly classified examples as assessed by Equation (8). The number of correct and wrong classification examples was denoted by (*num*_*c*_) and (*num*_*i*_), respectively.


(8) $$a\,c\,c=\frac{n\,u\,m_{c}}{n\,u\,m_{c}+n\,u\,m_{i}}\times 100\%$$

### Results and Discussion

We introduced the evaluation function in formula (7). The assessment used several well-known and recognized biomedical datasets (Hu and Dai, 2018). The datasets include ColonTumor and DLBCL-Outcome etc., and provide data related to gene expression, protein profiling, and genomic sequence for disease classification and diagnosis. All the datasets were high-dimensional and contained fewer instances and irrelevant or weak correlation features, the dimensional ranged from 2,000 to 12,600, and the format of the datasets was shown in Table 7.

**TABLE 7:** The format of datasets.

Data set	Instances	Attributes	Classes	K-NN (k = 5)	C4.5
ColonTumor	62	2,000	2	73.87 (0.24)	73.87 (0.24)
DLBCL-Outcome	58	7,129	2	47.46 (0.51)	47.46 (0.51)
ALL-AML-Leukemia	106	7,130	2	88.39 (0.13)	88.39 (0.13)
Lung cancer-Ontario	39	2,880	2	56.38 (0.34)	56.38 (0.34)
DLBCL-Stanford	47	4,026	2	75.51 (0.26)	75.51 (0.26)
Lung cancer-Harvard2	181	12,534	2	94.38 (0.04)	94.38 (0.04)
Nervous-System	60	7,129	2	54.63 (0.42)	54.63 (0.42)
Lung cancer-Harvard1	203	12,600	5	87.56 (0.09)	87.56 (0.09)
DLBCL-NIH	160	7,400	2	47.23 (0.46)	47.23 (0.46)

To evaluate the performance of our proposed BF-SFLA algorithm, the SFLA, the improved GA (IGA) (Yang et al., 2008), and the improved PSO (IPSO) (Chuang et al., 2008) were selected for comparison. In the experiments, consistent conditions and parameters were used in the comparative analysis, where the population size was 200 and the number of iterations was 500; the classification accuracy of feature subsets was evaluated using *K*-*NN* and *C*4.5 classification algorithms. In the BF-SFLA and the SFLA, (m) and (n) values were set to 5 and 5, respectively.

The training and the test samples should be independent to prove the generalization capability. In the experimentation, we used 10-fold cross-validation to estimate the classification rate for each dataset. These data were divided into 10 folds. For the 10 folds, 9 folds constitute the training set. The rest of the folds were used as the test set.

To avoid deviation, all results were the average of 30 independent executions of the algorithm. The aims were to reduce the number of feature subsets of datasets to less than 100 and improve the classification accuracy of the datasets. Nine typical high-dimensional biomedical data sets were selected, as shown in Table 7. The column titled *K-NN* and C4.5 represented the original data set’s classification accuracy, and the parentheses’ data expressed the average absolute error. In Table 8, nine datasets and four comparison algorithms were listed. Each algorithm had six attributes, which were i) the average fitness (Ave%), ii) the highest fitness (Max), iii) the lowest fitness (Min%), iv) the standard deviation (std), v) the average number of feature subsets (AveN), and vi) the number of algorithm executions in each experiment (S).

**TABLE 8:** The running result for four algorithms.

Data set	Algorithm	Ave(%)	Max(%)	Min(%)	Std	AveN	S
ColonTumor	**BF-SFLA**	**93.12**	**95.66**	90.23	**2.67**	**33.12**	6
	SFLA	89.02	91.66	**85.02**	2.69	36.16	6
	IGA	86.67	88.33	83.33	2.36	38.24	6
	IPSO	87.67	91.67	85.01	3.65	49.40	6
DLBCL-outcome	**BF-SFLA**	**74.23**	**77.63**	**67.21**	**3.26**	**26.25**	8
	SFLA	69.21	75.20	65.33	3.84	51.43	8
	IGA	64.33	70.06	60.00	5.21	27.62	8
	IPSO	71.11	76.67	63.33	5.34	51.24	8
ALL-AML-leukemia	**BF-SFLA**	**98.42**	**100.00**	**98.02**	**0.86**	**29.23**	8
	SFLA	97.27	99.09	94.52	1.93	45.65	8
	IGA	95.09	97.27	92.73	1.65	30.63	8
	IPSO	99.01	100.00	98.18	1.04	113.5	8
LungCancer-ontario	**BF-SFLA**	**75.55**	**80.12**	**71.67**	**3.24**	**14.65**	8
	SFLA	70.22	85.12	62.54	4.84	18.46	8
	IGA	65.51	75.21	57.52	4.18	10.22	8
	IPSO	70.00	77.50	57.50	4.89	56.25	8
DLBCL-stanford	**BF-SFLA**	**82.44**	**83.26**	**78.13**	**2.24**	**15.87**	8
	SFLA	80.01	82.01	78.04	2.06	25.67	8
	IGA	78.80	84.02	72.02	4.83	18.43	8
	IPSO	78.10	80.02	74.11	3.19	49.50	8
LungCancer-Harvard2	**BF-SFLA**	**98.94**	**99.65**	**97.45**	**0.98**	**51.87**	8
	SFLA	98.02	98.81	96.66	1.06	75.25	8
	IGA	96.67	98.33	95.56	1.11	52.80	8
	IPSO	96.36	99.98	93.34	2.33	98.31	8
Nervous-system	**BF-SFLA**	**81.75**	**85.26**	**78.13**	**3.34**	**32.24**	8
	SFLA	76.08	80.05	71.67	3.64	57.86	8
	IGA	71.67	81.67	61.67	7.16	30.25	8
	IPSO	72.67	78.33	63.33	6.07	45.03	8
LungCancer-harvard1	**BF-SFLA**	90.03	91.12	88.49	**1.11**	**28.24**	9
	SFLA	**91.21**	**92.24**	**89.22**	1.23	54.71	9
	IGA	85.90	87.50	84.09	1.29	31.81	9
	IPSO	91.90	94.14	90.04	1.51	44.20	9
DLBCL-NIH	**BF-SFLA**	**55.36**	56.84	**52.52**	**2.09**	**28.31**	8
	SFLA	54.16	**58.12**	50.63	3.13	30.75	8
	IGA	56.02	61.24	51.78	3.66	32.12	8
	IPSO	55.11	65.02	47.51	9.01	35.11	8

*The best value is in bold.*

As could be seen from Table 8, the BF-SFLA achieved the best Avg result among the four algorithms for eight of the nine data sets and the second best (Ave%) of the remaining dataset. The (Ave%) results for ColonTumor, DLBCL-Outcome, ALL-AML-Leukemia, Lung cancer-Ontario, DLBCL-Stanford, LungCancer-Harvard2, Nervous-System, and DLBCL-NIH obtained by the BF-SFLA were 93.12, 74.23, 98.42, 75.55, 82.44, 98.94, 81.75, and 55.36%, respectively. For the Lung cancer-Harvard1 dataset, the (Ave%) of BF-SLA was 90.03% while the SFLA obtained the best (Ave%) at 91.21%; however, the (AveN) for the SFLA dataset was 54.71, which was much larger than the BF-SFLA.

According to the (AvgN), the BF-SFLA obtained the minimum (AvgN) for all datasets compared with the SFLA, IGA, and IPSO algorithms. We could also observe that the standard deviation (Std) metric for all four algorithms in five of the nine data sets (as obtained by the BF-SFLA) was smaller than those of the other three evaluation algorithms. The best attribute results were shown in bold font in Table 8.

Table 9 showed the three average attribute values of AVE(Ave), AVE(Std), and AVE(AveN) for the nine datasets using the four algorithms for evaluation. Through comparative analysis of BF-SFLA with SFLA, IGA, and IPSO, BF-SFLA showed better performance improvement in classification accuracy and stability while using fewer relevant feature subsets. It could also be observed that due to the introduction of the proposed improvements and updating strategy, the BF-SFLA explored possible subsets space to obtain a set of features that maximize the predictive accuracy and minimize irrelevant features in high-dimensional biomedical data.

**TABLE 9:** The average attributes value for nine datasets.

Attributes	BF-SFLA	SFLA	IGA	IPSO
AVE(Ave)	**83.31**	80.57	77.55	80.21
AVE(Std)	**2.19**	2.60	3.49	4.11
AVE(AveN)	**28.86**	43.99	30.23	60.28

*The best value is in bold.*

**FIGURE 5:**
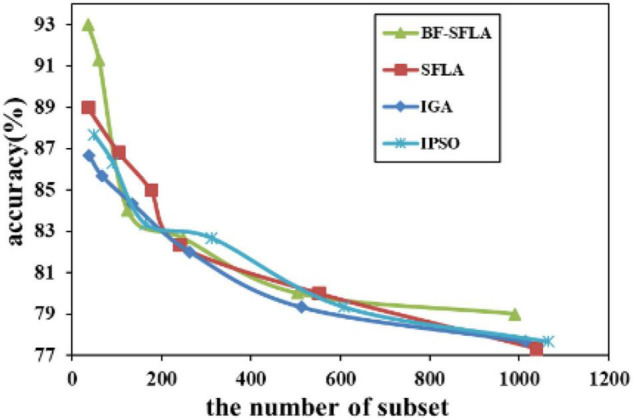
The variation trend of classification accuracy and feature subset of ColonTumor.

**FIGURE 6:**
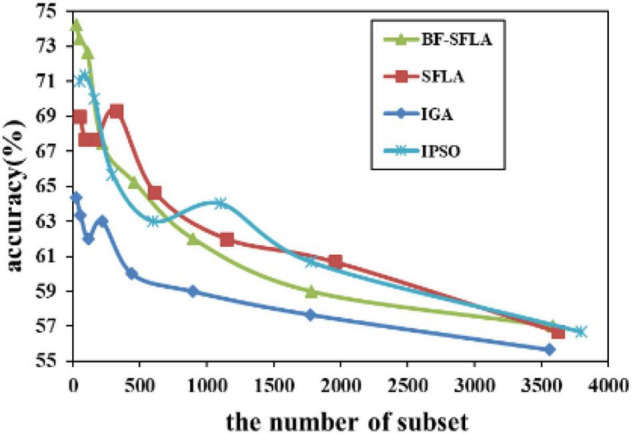
The variation trend of classification accuracy and feature subset e of DLBCL-Outcome.

**FIGURE 7:**
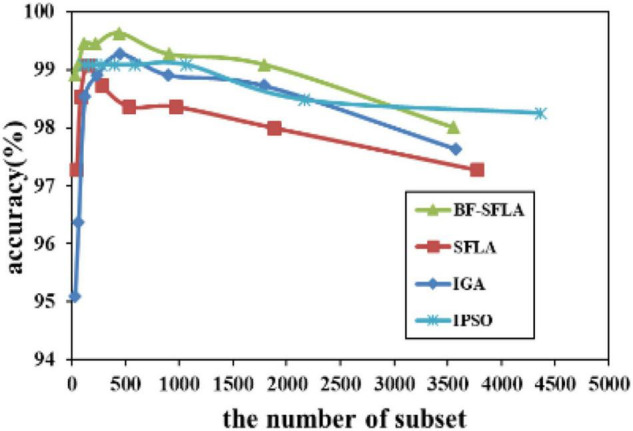
The variation trend of classification accuracy and feature subset of ALL-AML-Leukemia.

**FIGURE 8:**
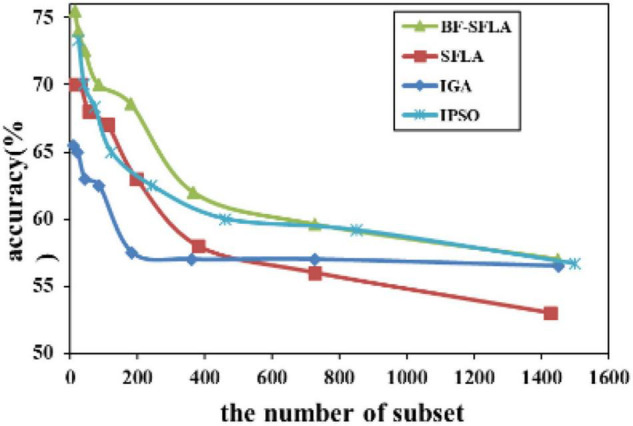
The variation trend of classification accuracy and feature subset of LungCancer-Ontario.

**FIGURE 9:**
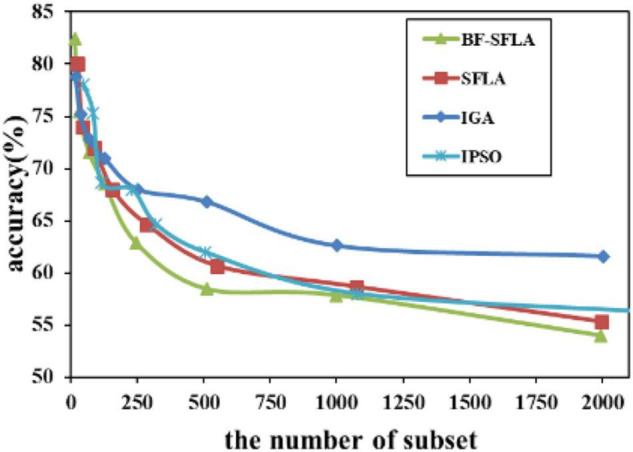
The variation trend of classification accuracy and feature subset of DLBCL-Stanford.

**FIGURE 10:**
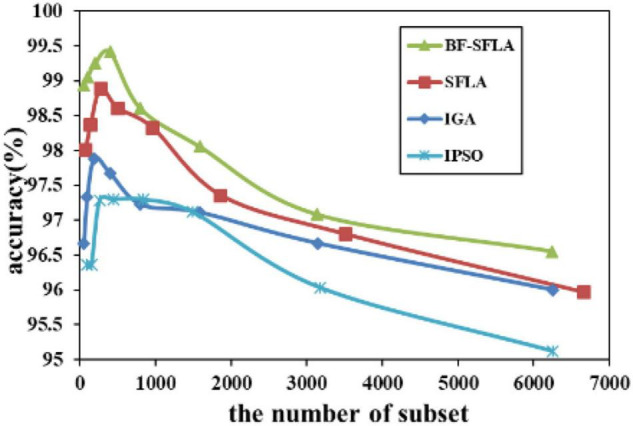
The variation trend of classification accuracy and feature subset of LungCancer-Harvard2.

**FIGURE 11:**
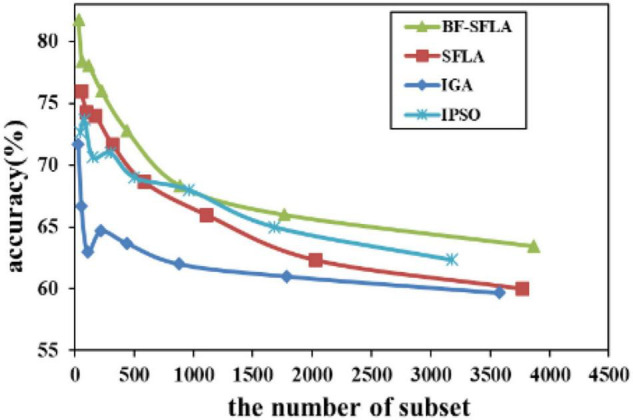
The variation trend of classification accuracy and feature subset of Nervous-System.

**FIGURE 12:**
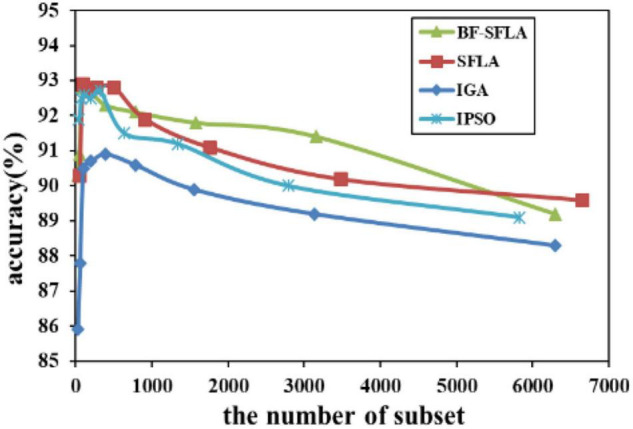
The variation trend of classification accuracy and feature subset of lungcancer-harvard1.

**FIGURE 13:**
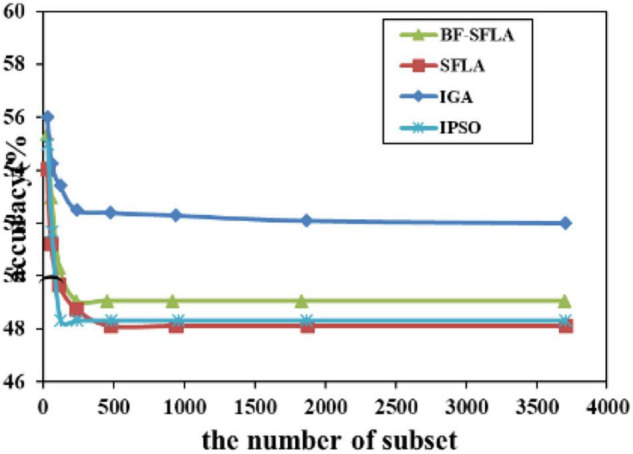
The variation trend of classification accuracy and feature subset of DLBCL-NIH.

The process of reducing the average value of feature subsets were shown in Figures 5–13. In each graph, the abscissa represented the number of feature subsets, and the ordinate represented the average classification accuracy of each algorithm executed 30 times independently. Figures 5–13 presented a performance comparison between the BF-SFLA and the SFLA, IGA, and IPSO methods. Figures 5, 6, 9, 13 showed that although there was no apparent advantage in the early-to-middle stages, the BF-SFLA algorithm could identify fewer feature subsets with higher classification effects and better performance later. Considering Figures 5–13 and Tables 8, 9, we discovered that the proposed improvements and updating strategy played a vitally important role in the feature selection performance of the BF-SFLA. It was worth noting that the purpose of feature selection was to move non-productive features without reducing the accuracy of prediction; otherwise, although the feature subset was small, the performance might be degraded. For example, for Figures 7, 10, 12, the average classification accuracy decreased gradually with the reduction of the number of features; therefore, we must balance the relationship between classification accuracy and the number of feature subsets in “real-world” applications so that the biological datasets set played a more critical role in the diagnosis of disease and improve the effectiveness of disease diagnosis (Vergara and Estévez, 2014).

## Conclusion

Feature subset selection was an essential technique in many application fields, and different evolutionary algorithms were developed for different feature subset selection problems. In this manuscript, the BF-SFLA algorithm was used to solve the problem of feature selection. By introducing the chemotaxis factor of the BF, a new ISFLA (termed the BF-SFLA) was adopted to solve the problem of feature selection in high-dimensional biomedical data, and the *K*-*NN* and *C*4.5 were used as the evaluator index of the proposed algorithm.

The experimental results showed that this method could effectively reduce the number of dataset features and simultaneously achieve higher classification accuracy. The proposed method could be used as an ideal pre-processing tool to optimize the feature selection process of high-dimensional biomedical data, better explore the function of biological datasets in the medical field, and improve the efficiency of medical diagnostics.

## Data Availability Statement

The original contributions presented in the study are included in the article/supplementary material, further inquiries can be directed to the corresponding author.

## Author Contributions

YD completed the overall experiment and wrote the first draft. LN normalized the data. LW and JT made grammatical modifications to the manuscript. All authors contributed to the article and approved the submitted version.

## Conflict of Interest

The authors declare that the research was conducted in the absence of any commercial or financial relationships that could be construed as a potential conflict of interest.

## Publisher’s Note

All claims expressed in this article are solely those of the authors and do not necessarily represent those of their affiliated organizations, or those of the publisher, the editors and the reviewers. Any product that may be evaluated in this article, or claim that may be made by its manufacturer, is not guaranteed or endorsed by the publisher.
